# The Value of Clinical Variables and the Potential of Longitudinal Ultrasound Carotid Plaque Assessment in Major Adverse Cardiovascular Event Prediction After Uncomplicated Acute Coronary Syndrome

**DOI:** 10.3390/life15030431

**Published:** 2025-03-09

**Authors:** Leonid L. Bershtein, Alexey N. Sumin, Anna V. Kutina, Marina D. Lunina, Dmitrii S. Evdokimov, Tatyana V. Nayden, Viktoriya E. Gumerova, Igor N. Kochanov, Arkadii A. Ivanov, Svetlana A. Boldueva, Ekaterina D. Evdokimova, Elizaveta V. Zbyshevskaya, Alina E. Evtushenko, Vartan K. Piltakyan, Sergey A. Sayganov

**Affiliations:** 1 Department of Internal Medicine & Cardiology, Northwestern State Medical University named after I.I. Mechnikov, 191015 St. Petersburg, Russia; viktoriya.gumerova@szgmu.ru (V.E.G.); e.zbyshevskaya@szgmu.ru (E.V.Z.); sergey.sayganov@szgmu.ru (S.A.S.); 2 Laboratory of Comorbidity in Cardiovascular Disease, Federal State Budgetary Institution ‘Research Institute for Complex Issues of Cardiovascular Disease’, 650002 Kemerovo, Russia; an_sumin@mail.ru; 3 Laboratory of kidney physiology and water-salt metabolism, Sechenov Institute of Evolutionary Physiology and Biochemistry of the Russian Academy of Sciences, 194223 St. Petersburg, Russia; kutina_anna@mail.ru; 4 Department of Diagnostics, Northwestern State Medical University named after I.I. Mechnikov, Mechnikov, 191015 St. Petersburg, Russia; marina.lunina@szgmu.ru (M.D.L.); tatyana.naiden@szgmu.ru (T.V.N.); Department of Internal Medicine, Northwestern State Medical University named after I.I. Mechnikov, 191015 St. Petersburg, Russiasvetlana.boldueva@szgmu.ru (S.A.B.); katerina.resn_7@mail.ru (E.D.E.); Department of Interventional Cardiology, Northwestern State Medical University named after I.I. Mechnikov, 191015 St. Petersburg, Russia; igor.kochanov@szgmu.ru (I.N.K.); arkadii.ivanov@szgmu.ru (A.A.I.); State Budgetary Healthcare Institution ‘Pokrovskaya City Hospital’, 199106 St. Petersburg, Russia; alinaevt0303@mail.ru (A.E.E.); vaite1984@icloud.com (V.K.P.)

**Keywords:** acute coronary syndrome, MACE prediction, carotid plaque, gray scale median

## Abstract

Due to the routine use of endovascular revascularization and improved medical therapy, the majority of acute coronary syndrome (ACS) cases now have an uncomplicated course. However, in spite of the currently accepted secondary prevention standards, the residual risk of remote major adverse cardiovascular events (MACEs) after ACS remains high. Ultrasound carotid/subclavian atherosclerotic plaque assessment may represent an alternative approach to estimate the MACE risk after ACS and to control the quality of secondary prevention. **Aim**: To find the most important clinical predictors of MACEs in contemporary patients with predominantly uncomplicated ACS treated according to the Guidelines, and to study the potential of the longitudinal assessment of quantitative and qualitative ultrasound carotid/subclavian atherosclerotic plaque characteristics for MACE prediction after ACS. **Methods:** Patients with ACS, obstructive coronary artery disease (CAD) confirmed by coronary angiography, and carotid/subclavian atherosclerotic plaque (AP) who underwent interventional treatment were prospectively enrolled. The exclusion criteria were as follows: death or significant bleeding at the time of index hospitalization; left ventricular ejection fraction (EF) <30%; and statin intolerance. The clinical variables potentially affecting cardiovascular prognosis after ACS as well as the quantitative and qualitative AP characteristics at baseline and 6 months after the index hospitalization were studied as potential MACE predictors. **Results**: A total of 411 primary patients with predominantly uncomplicated ACS were included; AP was detected in 343 of them (83%). The follow-up period duration was 450 [269; 634] days. MACEs occurred in 38 patients (11.8%): seven—cardiac death, twenty-five—unstable angina/acute myocardial infarction, and six—acute ischemic stroke. In multivariate regression analyses, the most important baseline predictors of MACEs were diabetes (HR 2.22, 95% CI 1.08–4.57); the decrease in EF by every 5% from 60% (HR 1.22, 95% CI 1.03–1.46); the Charlson comorbidity index (HR 1.24, 95% CI 1.05–1.48); the non-prescription of beta-blockers at discharge (HR 3.24, 95% CI 1.32–7.97); and a baseline standardized AP gray scale median (GSM) < 81 (HR 2.06, 95% CI 1.02–4.19). Among the predictors assessed at 6 months, after adjustment for other variables, only ≥ 3 uncorrected risk factors and standardized AP GSM < 81 (cut-off value) at 6 months were significant (HR 3.11, 95% CI 1.17–8.25 and HR 3.77, 95% CI 1.43–9.92, respectively) (for all HRs above, all *p*-values < 0.05; HR and 95% CI values varied minimally across regression models). The baseline quantitative carotid/subclavian AP characteristics and their 6-month longitudinal changes were not associated with MACEs. All predictors retained significance after the internal validation of the models, and models based on the baseline predictors also demonstrated good calibration; the latter were used to create MACE risk calculators. **Conclusions**: In typical contemporary patients with uncomplicated interventionally treated ACS, diabetes, decreased EF, Charlson comorbidity index, non-prescription of beta-blockers at discharge, and three or more uncontrolled risk factors after 6 months were the most important clinical predictors of MACEs. We also demonstrated that a lower value of AP GSM reflecting the plaque vulnerability, measured at baseline and after 6 months, was associated with an increased MACE risk; this effect was independent of clinical predictors and risk factor control. According to our knowledge, this is the first demonstration of the independent role of longitudinal carotid/subclavian AP GSM assessment in MACE prediction after ACS.

## 1. Introduction

In recent years, cardiovascular disease (CVD) has again become the leading cause of mortality in the United States [1], and in low- and middle-income countries the CVD mortality is 2–5 times higher than in more economically developed countries [2]. Coronary artery disease (CAD) accounts for the majority of cardiovascular deaths. The age-standardized CAD mortality rate per 100,000 is 108.8 globally, which is 2.5 times higher than that of ischemic stroke, the second most frequent cause of cardiovascular death [2]. The widespread use of early percutaneous coronary interventions (PCI) and the success of medical treatment have led to a decrease in the incidence of extensive left ventricular (LV) damage, early fatal arrhythmic events, in-hospital mortality, and the development of heart failure in the long term among contemporary patients with acute coronary syndrome (ACS) [3]. Despite this, the development of ACS increases the risk of recurrent atherothrombotic major adverse cardiac events (MACEs) in a patient with CAD in the long term by an average of an additional 50% [4]. In this regard, the search for the most important predictors of such events among patients with uncomplicated ACS treated according to contemporary standards seems relevant.

The reason for the development of recurrent MACEs after ACS is the failure to achieve the stabilization of atherosclerosis despite the Guidelines-based secondary prevention measures. Currently, the control of several major traditional atherosclerosis risk factors (TRF) is used as a criterion for the adequacy of the secondary prevention, and its targets are becoming more stringent in newer Guidelines [5,6]. Despite this, even among the participants of randomized clinical trials (RCTs), the recurrent MACE rate after ACS was 7–9% within 1 year [7], and remained high even with optimal TRF control [8].

At the same time, the studies in patients with carotid atherosclerosis showed that its variability was ‘explained’ by TRF only to a small extent—by about 20% [9]. Based on this, it can be assumed that in the case of coronary atherosclerosis, even the best TRF control does not warrant the stabilization of atherosclerosis (apparently, the opposite situation is also possible).

Vascular imaging is an alternative approach to assess atherosclerosis stabilization. One of its methods, ultrasound carotid study, does not result in radiation exposure, has good reproducibility, and provides a way to track changes in the variables related to atherosclerotic burden and atherosclerotic plaque (AP) composition during the cholesterol-lowering therapy [10,11].

Coronary atherosclerosis severity is known to be an important prognostic factor in CAD [12]. We hypothesized that, given the certain extent of parallelism of the atherosclerotic process in the carotid and coronary arteries [13], the baseline carotid atherosclerosis variables after ACS, which directly reflect the atherosclerotic burden in another vascular territory, may have an added prognostic value to the assessment of coronary disease anatomic severity and be more accurate predictors of recurrent MACEs than the traditionally used clinical variables. Apart from the atherosclerotic burden, ultrasound carotid study also allows for the indirect assessment of the plaque composition, which reflects its stability [14]—such information regarding the coronary plaque cannot be obtained from routine coronary angiography and requires the utilization of sophisticated intravascular imaging methods [15]. The relationship between quantitative and qualitative carotid plaque variables and cardiovascular prognosis was previously shown in primary prevention studies [16,17].

Moreover, longitudinal changes in carotid atherosclerosis variables, including those in the coronary bed, may reflect the changes in the atherosclerotic process better than the extent of risk factor control, which is the basis of the current secondary prevention approach.

These data were a rationale for studying ultrasound carotid plaque variables measured at baseline and at follow-up as potential MACE predictors in patients after ACS.

It was also essential to know which of the clinical variables remained important predictors of prognosis in ACS in the modern era.

**The aim of this study** was to find the most important clinical predictors of MACE in contemporary patients with predominantly uncomplicated ACS receiving treatment according to clinical Guidelines, and to study the potential of the longitudinal assessment of quantitative and qualitative ultrasound carotid/subclavian atherosclerotic plaque characteristics for MACE prediction after ACS.

## 2. Methods

The study protocol was approved by the Local Ethics Committee of the Northwestern State Medical University named after I.I. Mechnikov. Before enrollment, the patients signed the informed consent form according to the standard procedure. The patients were recruited in two large vascular centers: one within the University and another outside of it.

### 2.1. Selection Criteria

The inclusion criteria were as follows:

- ACS of any type; obstructive CAD confirmed by coronary angiography; primary invasive strategy (PCI).
- Evidence of atherosclerotic plaque at carotid/subclavian ultrasound study.

The diagnosis of ACS was made based on the standard criteria [18,19]. Obstructive CAD was confirmed by the presence of at least one narrowing of >50% in the main epicardial arteries or their large branches detected at coronary angiography. During the index hospitalization, clinical, laboratory, electro- and echocardiographic, and angiographic parameters—potential recurrent MACE predictors—were registered.

The search for atherosclerotic plaques was performed in the extracranial sections of the carotid arteries, as well as in the right subclavian artery. Atherosclerotic plaque was defined according to the Mannheim consensus criteria [20].

The exclusion criteria were as follows:

- Death or clinically significant bleeding at index hospitalization.
- Left ventricular ejection fraction (EF) <30% and/or heart failure NYHA class IV at hospital discharge.
- Planned coronary artery bypass surgery (CABG).
- Statin intolerance.
- Severe comorbidity with a life expectancy of less than 1 year.
- Stenotic calcific carotid lesion precluding quantitative atherosclerotic plaque analysis.

Since the cardiovascular outcomes we studied were those related to atherothrombotic events, the patients with a high risk of death from other causes were not enrolled. This was the reason for the exclusion of patients with a low EF, in whom the primary causes of death were ventricular arrhythmia (not related to new atherothrombosis) and heart failure [21]. Moreover, our intention was to assess the typical contemporary ACS patients who predominantly have preserved EF [3]. The patients scheduled for CABG as a primary revascularization method were excluded due to the significant difference in their clinical profile compared to the majority of ACS patients eligible for PCI. The proportion of patients with ACS requiring primary CABG is known to be 10% [22,23].

### 2.2. Goals of Revascularization and Medical Therapy

The scope of revascularization was determined by interventional cardiologists. In the absence of contraindications, the goal was to perform complete revascularization. Second-generation drug-eluting stents were universally used.

Medical therapy was prescribed by cardiologists according to the usual standards. The goal was the prescription of optimal medical therapy (OMT), including, as a rule, dual antiplatelet therapy, a beta-blocker, a renin–angiotensin–aldosterone system (RAAS) inhibitor, and a high-intensity statin, if necessary, in combination with ezetimibe. The goal of OMT was to achieve control of the most important TRFs: blood pressure (BP), low-density lipoprotein cholesterol (LDL-C cholesterol), glycosylated hemoglobin in diabetes mellitus (DM), smoking, and physical inactivity, in accordance with current Guidelines [18,19,24,25,26].

### 2.3. Ultrasound Study Protocol

The study of the extracranial sections of the carotid arteries was performed in B-mode using an expert-class Philips Affiniti 50 (Netherlands) ultrasound scanner with a linear 5–12 MHz transducer. Color Doppler blood flow mapping and spectral Doppler study were performed optionally. The far and near walls of the common carotid artery (CCA), CCA bifurcation, and the internal carotid artery were examined along the entire length accessible for ultrasound scanning in longitudinal, anterior, and lateral planes, as well as in cross section. Due to the anatomy of the left subclavian artery, which branches directly from the aortic arch behind the left clavicle, the ultrasound assessment of its origin is often not feasible. For this reason, only the right subclavian artery assessment was performed, for which, if necessary, a convex 2–5 MHz transducer was additionally used. The evaluation of the right subclavian artery, in most cases not included in the standard quantitative ultrasound analysis protocol, was considered necessary based on the data of Pescetelli et al. [25] on frequent subclavian plaque detection in the absence of carotid and coronary atherosclerosis in patients with a lower cardiovascular disease (CVD) risk.

The quantitative plaque analysis included the measurement of the AP height, area, and the degree of lumen stenosis by diameter and area according to the ESCT method [27]. The number of APs was counted. To measure the AP height, the projection where it was greatest was selected. The total height of the atherosclerotic plaques (Hsum) was measured as the sum of the maximum heights of all identified APs. The area of the atherosclerotic plaque was measured in one longitudinal plane and projection; the specialist selected the projection with the largest atherosclerotic plaque area. To calculate the area of the AP, it was traced along the outer border [10]. In the case of multiple plaques, all area values were summed up, and thus the total plaque area (TPA) was calculated.

In the pilot study [28] involving the same ultrasound specialists as in this study, the interobserver variability of carotid ultrasound measurements was higher than the intraobserver variability, which matched the previously reported results [11]. For this reason, we organized the follow-up ultrasound study to be, as a rule, performed by the physician who had performed the baseline exam. In the same pilot study [28], the intraobserver variability of the assessed variables was small (Hsum 0.10, 95% CI −0.23–0.44 mm; TPA 1.05, 95% CI −0.54–2.63 mm^2^); for comparison, the mean annual TPA change was previously shown to be about 3–4.6 mm^2^ [11] even in a primary prevention cohort, which allowed us to expect the reliable tracking of the longitudinal TPA changes in this study.

GSM analysis was performed using the standard methodology [29] for those APs in which it was technically possible. The standardized GSM value of the patient was calculated as the sum of the products of each GSM value by the area of the corresponding AP divided by the TPA, according to the previously described methodology [11]. When calculating the TPA for this purpose, only APs where the GSM analysis was performed were taken into account. Ultrasound images of plaques with different GSM values are presented in the Supplementary file (Figure S1).

### 2.4. Follow-Up and Registration of MACE

The 6-month visit was performed in person, if possible, and included a repeat ultrasound study according to the protocol described above, as well as the MACE registration. The current treatment and TRF control were registered. The registered MACE included cardiac death, hospitalized unstable angina (UA)/acute myocardial infarction (AMI) and acute ischemic stroke. Only MACEs that occurred after the end of hospitalization for the index ACS event were registered. Visits at 12, 24, 36 months and the final visits were performed by phone contact, and the same clinical information was collected. According to the Guidelines [18,19], TRF control was defined as follows: for blood pressure (BP):achieving a systolic BP of 120–130 mm Hg or lower (for individuals over 80 years old—130–140 mm Hg or lower) and a diastolic BP of 70–80 mm Hg or lower; for low-density lipoprotein cholesterol (LDL-C) level: <1.4 mmol/L *and* decrease of ≥ 50% from baseline; for physical activity: achieving a moderate activity level for >150 min *or* vigorous activity level for >75 min per week; and smoking cessation.

**Figure 1:**
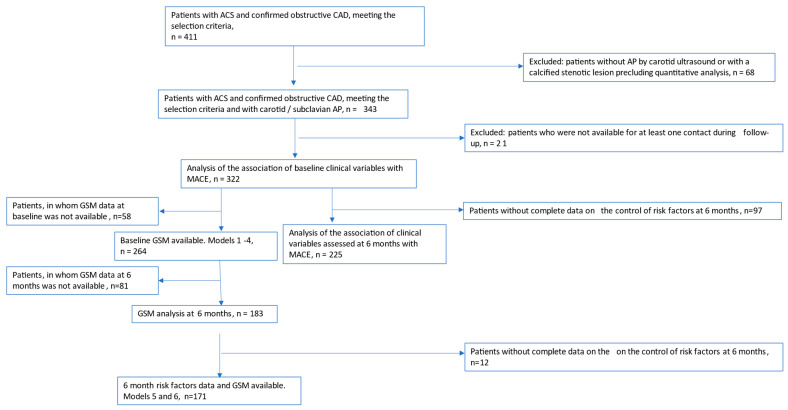
CONSORT flow diagram. Flowchart showing the inclusion and numbers of patients available for the most important analyses throughout the study. Note: The total number of patients meeting the selection criteria and having carotid/subclavian plaque was 343, of whom 21 were completely lost to follow-up. In 322 patients, the univariant regression analysis of the baseline clinical variables association with MACEs was conducted. Baseline GSM analysis data were available in 264 patients. Their data were used for creating multivariant regression models 1–4 (32 outcome events). After 6 months of follow-up, 97 patients had no full data on the risk factor control; univariant regression analysis of the clinical variables at 6 months and their association with MACE was performed in 225 patients. The number of patients in whom GSM data at 6 months was not available was 81; GSM analysis at 6 months was performed in 183 patients. Since complete data on the control of risk factors at 6 months was not available in 12 of these patients, multivariant regression models 5 and 6 were created on the data from the 171 remaining patients (18 outcome events).

The study flowchart with the numbers of patients available for the most important analyses are presented in the CONSORT diagram (Figure 1).

### 2.5. Statistical Analysis

The analysis was performed using the STATA 8.0 software package (StataCorp LLC, College Station, TX, USA). The Shapiro–Wilk test was used to test the normality of the quantitative variable distribution. Since almost all quantitative variables did not correspond to the normal distribution, nonparametric statistics were used. The absolute number of observations, the proportions for the categorical variables, and the median and interquartile range (Q1; Q3) for the ordinal and quantitative variables were used to describe the data.

For survival analysis, the Kaplan–Meier method was used to calculate and display the incidence of MACEs (as a hazard cumulation function) in the patient cohort. Time was calculated from the date of hospitalization for ACS to the date of the MACE or the date of last contact with the patient (routine visits were carried out 6, 12, and 24 months after discharge from hospital). The probability of developing a MACE at 6, 12, and 24 months of follow-up was estimated with 95% confidence intervals (CI). The type of missing data in all variables was missing completely at random.

Univariate Cox regression analysis was first performed to screen for the potential risk factors associated with MACEs. Standardized GSM was converted to a binary variable; the cut-off value of ≤81 was chosen based on the highest significance of the association of the obtained variable with MACEs in univariate regression. Independent variables that showed a nonadjusted effect on MACEs with *p* < 0.15 in the univariate analysis were subjected to multivariate Cox proportional hazards regression analysis with a stepwise selection procedure. Complete case analysis was used for multivariable regressions. Significance levels for the inclusion of variables in the model were tested based on the likelihood ratio (exclusion threshold *p* = 0.1). Model development and reporting followed the TRIPOD guidelines [29]. When forming the initial set of independent variables, we avoided the simultaneous inclusion in the analysis of predictors associated with each other (Spearman’s rank correlation coefficient >0.7; statistically significant association identified by logistic regression analysis), as well as clinical prognostic scores and the variables underlying their assessment. As a result, six multivariate models were built based on different initial sets of independent variables. The proportional hazards assumption was checked using Schoenfeld residuals after the fitting of each model. Hazard ratios (HR) in univariate regression analysis and adjusted hazard ratios in multivariate regression analysis were calculated with a 95% CI. The predictive performances of each multivariate model were quantified and compared on the basis of the Akaike information criterion (AIC) and the Bayesian information criterion (BIC). AIC and BIC were calculated based on the model’s log-likelihood and the number of parameters and allowed to compare models by the best fit to complexity ratio. When comparing models, the lower the AIC or BIC, the better the model. The predictive accuracy of the models was further evaluated using Harrell’s concordance index (C-index). The C-index is a measure of a model’s discrimination ability and can be interpreted as the rank correlation between the predicted probabilities of the outcome occurring and the observed response. A higher value of C-index implies a greater prediction performance associated with the regression model, with a maximum possible value of 100%. In addition, the availability and clinical significance of the predictors were assessed. The goodness-of-fit and calibration of the obtained models were assessed by plotting the Cox–Snell residuals against the cumulative hazard function and by the Groennesby and Borgan test. A model was well calibrated when the test yielded no significant differences (*p*-value > 0.05; the higher the *p*-value, the better the calibration). The models were internally validated via the bootstrapping method, and the bootstrap-based (200 replicates) 95% CI for the adjusted HR for each parameter were calculated.

Based on multivariate Cox regression models that demonstrated high accuracy in terms of the information criteria (AIC) and (BIC), as well as good discrimination and calibration characteristics, calculators were created that allowed the estimation of the risk of MACEs in a patient at 0.5–2 years after the ACS. For this purpose, the HR for each significant predictor in the regression model was estimated, as well as the baseline cumulative hazard for time points of 180, 360, and 720 days, which were equal for all study subjects (provided that the values of independent quantitative variables were equal to a certain selected value, and the values of binary variables were those that did not affect the outcome). Individual risk was calculated by multiplying the baseline cumulative hazard and the effect of the predictors.

All statistical comparisons were considered significant at *p* < 0.05.

## 3. Results

Between 1 February 2022 and 3 May 2024, 411 patients with ACS undergoing primary PCI were assessed for eligibility; among them, carotid/subclavian AP available for quantitative analysis was detected in 343 (83%) (Figure 1). The last follow-up visit was conducted in October 2024.

### 3.1. Variables Assessed at Baseline and 6 Months After Index ACS and the Risk of MACEs

The follow-up duration was 450 [269; 634] days, with a maximum follow-up time of 800 days. MACEs occurred in 38 patients (11.8%): seven—cardiac death, twenty-five—UA/AMI, and six—acute ischemic stroke; in patients in whom AP GSM at 6 months after ACS could be assessed as a predictor, 18 MACEs occurred (three—cardiac death, thirteen—UA/AMI, and two—acute ischemic stroke).

**Figure 2:**
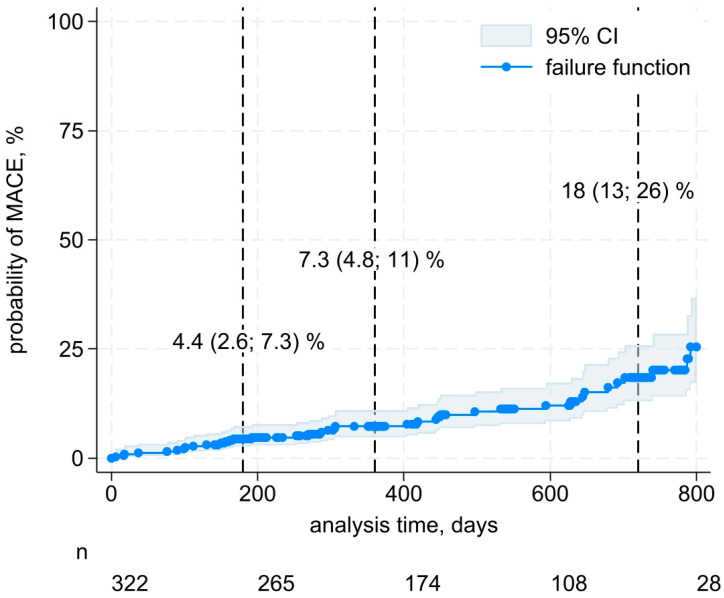
Kaplan–Meier curve of MACE probability as a function of the follow-up time. Shading indicates the 95% confidence band for MACE probability. Note: The risk accumulation function is calculated for a maximum follow-up period of 800 days (median 450 [269; 634] days), and *n* is the number of patients at risk. The calculated MACE probability values (with 95% CI) are shown for 180, 360, and 720 days of follow-up.

Figure 2 shows the MACE risk accumulation function in our study.

The general characteristics of the study patients assessed at baseline and after 6 months of follow-up by MACE occurrence are given in Table 1 and Table 2.

The participants’ median age was 65 [59; 73] years, and 69.6% were males. Smoking history was present in 46.9%, 20.8% had diabetes, and 28% had a history of AMI. Left main disease was found in 11.5%, and three-vessel disease in 28.7% of patients. The proportion of NSTE-ACS was 47.3%. The median LV ejection fraction was not reduced, being 61 [54; 66] %. A standardized GSM of less than 81 (cut-off value in our study) was detected in 33% of patients. A total of 42.2% of patients underwent complete revascularization at discharge. High-intensity statin was prescribed to 95.3% of patients at discharge.

At 6 months after ACS, complete adherence to OMT exceeded 90%. However, the correction of the main risk factors was not optimal. BP control was achieved most often (81%), while the recommended reduction in LDL-C was achieved in the minority of participants (12%). A total of 19% of patients had three to four uncorrected risk factors. An increase in TPA was noted in 29% of patients, while a decrease in the standardized AP GSM was found in only 5%.

In Table 3, variables that showed statistically significant or borderline nonadjusted effects on MACEs based on the results of the univariate Cox regression analysis are presented.

In univariate regressions, significant/borderline associations with MACEs were found for presence of diabetes, history of AMI, LV ejection fraction, three-vessel coronary artery disease, Charlson comorbidity index, heart rate at discharge, beta-blocker prescription at discharge, having three to four vs. zero to two uncontrolled risk factors at 6 months, and standardized carotid/subclavian AP GSM at baseline and after 6 months.

Based on the results of the univariate analyses and taking into account the presence of interrelated variables in the list of predictors (Charlson comorbidity index and diabetes, history of myocardial infarction and stroke, heart rate at discharge, and prescription of beta-blockers), four models with different initial sets of variables for selection were constructed. These models are presented below in the original version and after internal validation (Table 4).

After adjusting for other variables, the most important predictors of MACE were DM, EF, Charlson comorbidity index, non-prescription of a beta blocker at discharge, and standardized GSM < 81.

The comparison of models 1–4 by AIC, BIC, and Harrel’s C-index is presented in Table S1, while the calibration results are shown in Figure S2 (Supplementary file). The diagnostic value of all models was similar, and the predictors in almost all cases retained their significance after internal validation. The calibration of all models was good.

Table 5 presents the results of the multivariate Cox regression analyses with the addition of the most significant baseline variables to those variables assessed at 6 months that were significantly associated with MACEs in univariate regression.

Among the predictors assessed at 6 months, ≥3 uncorrected risk factors and standardized GSM < 81 were the only significant predictors after adjusting for other variables.

The comparison of models 5 and 6 by AIC, BIC, and Harrel’s C-index is presented in Table S2, while the calibration results are shown in Figure S3 (Supplementary file). As for models 1–4, their diagnostic value was similar, and most predictors retained their significance after internal validation. However, though model 6 passed the calibration according to the Grønnesby–Borgan test, the calibration of this model on goodness-of-fit graphs was unsatisfactory.

### 3.2. Calculators Based on the Identified Predictors

The individual risk calculators were created based on models 1–4, which have demonstrated good calibration results.

The first calculator was based on models 1–2, in which the MACE predictors were diabetes (HR 2.22 and 2.28), LV EF (HR 1.22 and 1.24), and baseline standardized GSM (HR 2.06 and 1.98). The second calculator was based on models 3–4, in which the MACE predictors were the Charlson comorbidity index (HR 1.22 and 1.27), LV EF (HR 1.22 and 1.23), and baseline standardized GSM (HR 2.01 and 1.97). The HR values for the listed predictors in models 1 and 2 were almost identical to those in models 3 and 4, but models 2 and 4 allowed for the adjustment for differences in treatment when calculating the baseline risk. The calculation of the baseline risk was performed for the value of the variable ‘No beta-blockers at discharge’—since, according to current Guidelines, beta-blockers after ACS are a class I (EF < 40%) or IIa (all patients) recommendation [30].

Table 6 shows the baseline (with optimum values of predictors) cumulative MACE risk (BCR) at different timepoints after ACS.

### 3.3. Equations for Calculating Individual Risk

#### 3.3.1. Calculator 1

Individual risk = BCR ∗ 2.28 (if the person has treated diabetes) ∗ 1.98 (if the baseline standardized GSM is ≤81) ∗ 1.24 ^n^, where n = (60—LV EF (in %) of the patient)/5.

The calculation examples are as follows:

**Example** **1.** *In a patient without DM, with LV EF = 40% and standardized GSM = 70, the risk of MACE at 360 days can be calculated as follows:*

3.35% ∗ 1.98 ∗ 1.24 ^4^ = 3.35% ∗ 1.98 ∗ 2.36 = 15.7%, since n = (60–40)/5 = 4.

**Example** **2.** *In a patient with treated DM, LV EF = 40% and GSM = 70, the risk of MACE at 360 days can be calculated as follows:*

3.35% ∗ 2.28 ∗ 1.98 ∗ 1.24 ^4^ = 3.35% ∗ 2.28 ∗ 1.98 ∗ 2.36 = 35.7%, since n = (60–40)/5 = 4.

#### 3.3.2. Calculator 2

Individual risk = BCR ∗ 1.27 ^m^ ∗ 1.97 (if the baseline standardized GSM is ≤81) ∗ 1.23 ^n^, where m = Charlson index, n = (60—LV EF (in %) of the patient)/5.

The calculation examples are as follows:

**Example** **3.** *If a patient has a Charlson comorbidity index of 8 with LV EF = 40% and standardized GSM = 70, the risk of MACE at 360 days can be calculated as follows:*

1.52% ∗ 1.27 ^8^ ∗ 1.97 ∗ 1.23 ^4^ = 1.52% ∗ 6.77 ∗ 1.97 ∗ 2.29 = 46.4%,

*because m = 8 and n = (60*–*40)/5 = 4.*

**Example** **4.** *If a patient has a Charlson comorbidity index of 2 with LV EF = 55% and standardized GSM = 90, the risk of MACE at 360 days can be calculated as follows:*

1.52% ∗ 1.27 ^2^ ∗ 1.23 ^1^ = 1.52% ∗ 1.61 ∗ 1.23 = 3.0%,

*because m = 8, and n = (60*–*55)/5 = 1.*

## 4. Discussion

We prospectively studied typical contemporary ACS patients who were predominantly without severe LV dysfunction or acute heart failure, who were treated with PCI and OMT, and who had concomitant carotid/subclavian atherosclerosis.

The overall incidence of cardiovascular events during a median of 1.2 years of follow-up in patients with ACS and carotid atherosclerosis was 11.8%, including a mortality rate of 2.2%. These figures were obtained in patients discharged from the hospital, i.e., they did not include in-hospital non-fatal events and mortality. For comparison, in a large Finnish registry of an earlier period (1993–2011) which also assessed the events starting from day 28 after ACS, the incidence of recurrent MACEs was 34.4%, including a mortality rate of 12.2% within 1 year [31]. This apparently marks a decrease in the MACE incidence after ACS, even taking into account the exclusion of several high-risk patient categories (low LV ejection fraction, need for primary CABG, calcified stenotic carotid lesions) from our study.

We assessed the TRFs, clinical variables, electro- and echocardiographic variables reflecting the size of LV damage, and variables related to the severity of coronary and carotid atherosclerosis to find those having an independent effect on the MACE risk in the mid-term after the ACS.

For a number of potential risk factors (age, male gender, LDL-C, LDL-C/HDL-C ratio, etc.), no significant effect on MACEs was shown. Previous similar analyses were inconclusive. Bilgin et al. [32] found total cholesterol, triglycerides, and some metabolic indices based on these variables to be the independent MACE predictors after ACS, while Okkonen et al. [31] found them to be non-predictive after a 1-year follow-up. Fox et al. [33], in a follow-up of over 30,000 patients for up to 5 years, showed the moderate prognostic effect of age. LDL-C on admission may be lower in those with more aggressive treatment before the ACS due to the higher baseline risk; therefore, in our opinion, a reduction in MACE rates with lower LDL-C on admission should not be expected.

The prognostic role of hemodynamic variables (heart rate, systolic BP on admission) and hemoglobin level after ACS is well known [33]. A recent large study found that in-hospital bleeding has a significant adverse effect in the long-term [34]. However, we were predominantly interested in studying the remote prognosis prediction in patients with uncomplicated ACS, and therefore those with initial severe hemodynamic disturbances and major bleeding during ACS were usually not included, so the variability of these parameters in the enrolled patients was minimal and they did not show significant prognostic effects.

In the univariate analyses of the baseline variables, history of AMI, three-vessel coronary artery disease, diabetes, LV ejection fraction, Charlson comorbidity index, heart rate at discharge, and beta-blocker prescription at discharge had a significant effect on the prognosis of MACEs after ACS.

In multivariate regressions, the significant independent effects of these variables on MACE prognosis were confirmed, with the exception of the first two. In univariate and then multivariate analyses of the variables assessed at 6 months, uncontrolled risk factors (three to four vs. zero to two) had a significant effect on the MACE risk independent of the baseline variables.

We also managed to demonstrate the independent prognostic effect of the standardized carotid/subclavian AP GSM at baseline and after 6 months on MACE.

The data on the prognostic significance of severe coronary atherosclerotic burden on MACEs in the mid-term in ACS patients are less robust than in stable CAD. In previous studies of ACS, the relative independent prognostic significance of multivessel coronary artery disease was small [35]. In our study, three-vessel coronary artery disease was a predictor of MACEs, but its effect became insignificant when adjusted for other variables.

Variables reflecting the size of LV damage have previously demonstrated a significant impact on the long-term prognosis after ACS [36]. In recent years, higher EF values have been observed in patients with ACS, reflecting the successful use of early PCI and OMT [3]. This is also true for our study, where the median EF was not reduced (61%). However, EF remains the important determinant of prognosis after ACS [37]. In our study, LV ejection fraction was one of the few variables that had a significant impact on the incidence of MACEs, which was confirmed in multivariate regression. A 5% decrease in EF resulted in a MACE risk increase by 22–24%, depending on the model.

The prevalence of diabetes in patients with ACS is high—up to 25% among patients with STEMI [38]. In our study, 21% of patients had treated diabetes. It is known that patients with ACS combined with diabetes have a worse prognosis, which is due to a combination of several pathophysiological mechanisms, including insulin resistance, hyperglycemia, endothelial dysfunction, and platelet activation and aggregation, as well as more unfavorable characteristics of coronary atherosclerosis, including its greater severity [38,39]. In the study by Okkonen et al. [31], the presence of DM increased the risk of MACEs by 1.4 times within 1 year after ACS. In our study, diabetes was an even more powerful predictor, increasing the MACE risk by 2.2–2.3 times.

The Charlson comorbidity index is an integral variable that includes a history of AMI, as well as a number of the most important markers of high cardiovascular risk, including DM, peripheral arterial disease, and chronic kidney disease. In our study, an increase in the index by 1 point resulted in an increase in MACE risk by 24–27%, which is consistent with the data of previous studies [31]. It should be noted that a number of variables included in the Charlson index also influenced the development of MACEs, including a history of AMI and diabetes, which may partially explain its prognostic effect. Nevertheless, based on our results, routine Charlson index calculation in patients with ACS seems prudent.

The role of routine beta-blocker administration in contemporary patients with ACS with predominantly preserved EF who have undergone revascularization and who receive OMT is controversial. Observational studies have shown a potential role for beta-blockers in the prevention of MACEs, even in contemporary conditions [40], while the results of RCTs on this issue are expected soon and are likely to be negative [41]. The non-administration of beta-blockers was associated with worsening prognosis in our study, but it was not designed to assess the effects of treatments on the outcome, and therefore this result is not sufficiently reliable.

The influence of carotid/subclavian atherosclerotic burden/AP composition and their longitudinal changes on cardiovascular risk in primary prevention have been the subject of recent research [16,17], but their role after ACS is poorly studied. According to our data and some other sources, the majority of patients with ACS have subclinical carotid AP available for evaluation [42,43]. In a cross sectional analysis, we demonstrated a moderate correlation between the severity of coronary and carotid atherosclerosis [38], but in this prospective study we did not find a relationship between the baseline quantitative characteristics of carotid/subclavian AP as well as their changes after 6 months and MACE recurrence. Since the increase in coronary atherosclerotic burden is known to be a powerful predictor of coronary events [44], the lack of a relationship between carotid/subclavian AP progression and MACEs apparently indicates that the rates of atherosclerosis progression in different arterial beds do not correlate.

At the same time, we were able to demonstrate that another variable associated with carotid atherosclerosis—standardized GSM, reflecting the carotid plaque composition and ‘vulnerability’ [45]—is an independent determinant of predominantly cardiac MACEs, being valid even after adjustment for significant clinical predictors of prognosis. In particular, the presence of baseline standardized GSM < 81 (cut-off value) approximately doubled the risk of MACEs. This is consistent with the data of Futamata et al. [46], who showed that signs of carotid atherosclerosis instability correlated with the progression of coronary atherosclerosis—an important predictor of MACEs. We were able to directly demonstrate a MACE risk increase in the presence of more ‘vulnerable’ carotid AP by ultrasound criteria.

Similarly, MACE risk increased with lower GSM values at 6 months after ACS, as well as with less controlled TRFs. Risk factor control is an important determinant of prognosis after ACS. In the BARI 2 D study of patients with stable CAD, risk factor control 1 year after inclusion in the study was closely associated with survival and cardiovascular outcomes [35]. Similar results were obtained in our study. The number of uncontrolled major TRFs (LDL-C, BP, physical activity, smoking) at 6 months (three to four vs. zero to two) increased the risk of MACE by 2.3–3.1 times after adjusting for other variables.

In general, according to Harrel’s C-index, the prognostic value of the models based on variables assessed 6 months after ACS was higher than that of models based on the baseline variables, and the HR for GSM < 81 at 6 months was 3.8–4.0 independently of the TRF control. This result is consistent with the work of Frigerio B et al., who showed that in European patients with high cardiovascular risk, standard risk factors explained only 12% of the carotid GSM variability [47]. According to our results, even with good TRF control, lower carotid GSM values may persist during the first year after ACS, while the longitudinal carotid GSM evaluation after ACS can be used as an independent tool to predict the MACE risk. Also, 6-month carotid GSM may be suggested as a potential new marker of the secondary prevention adequacy—to prove this, further research is required.

A likely mechanism explaining the findings associated with carotid/subclavian AP GSM is the concept of ‘systemic plaque vulnerability’. According to this concept, destabilization of atherosclerosis is a systemic process that occurs in parallel in different arterial beds; in this case, the carotid and coronary [48]. Hence, the ultrasound signs of the carotid/subclavian AP vulnerability probably reflect the destabilization of atherosclerosis in the coronary bed as well, which results in an increased MACE rate.

The value of the prognostic models based on the listed predictors was confirmed by internal validation [29], and in the case of the baseline variables, by calibration. The good calibration results of models 1–4 allowed us to create two MACE risk calculators that included the baseline AP GSM value.

Currently, ultrasound carotid plaque assessment is not represented in the clinical guidelines as a part of routine in-hospital evaluation in ACS [18,19,30]. The method’s high reproducibility was previously reported [11,28], while according to this study’s results, it may be universally recommended as a simple non-invasive risk-assessment tool available in the majority of patients. Its use may result in identifying subjects with a higher probability of recurrent MACEs among the category of patients with uncomplicated ASC without high-risk clinical features, which is of undoubtful clinical importance.

*Study limitations*: Our study was performed on a relatively small group of patients recruited in two clinical centers. The duration of follow-up was relatively short, with a median of 1.2 years. A small proportion of patients (6%) were lost to contact, and we could not analyze the information on possible MACEs for them.

The GSM analysis we used requires ultrasound image storage with subsequent off-line TPA and GSM measurements and standardized GSM calculation, which is relatively time-consuming.

The longitudinal analysis of ultrasound carotid/subclavian AP variables could be performed only in a fraction of the included patients. Due to the significantly smaller number of patients in whom the follow-up GSM value was available compared to the baseline GSM data, the confidence intervals of HRs for the variables assessed at 6 months were relatively wide and the calibration of the model based on the GSM value after 6 months was unsatisfactory. So, we considered it inappropriate to create a risk calculator based on this variable, in spite of its strong independent effect on MACEs in multivariant regression models.

The algorithms including the data of ultrasound carotid/subclavian imaging that we proposed for determining the risk of MACEs after ACS were validated only in the sample of study participants and require external validation.

## 5. Conclusions

We studied typical contemporary patients with uncomplicated ACS and obstructive coronary artery disease. Concomitant carotid/subclavian atherosclerosis was found in 83%, so the approach to MACE prediction and possibly for secondary prevention control that we suggested is feasible in the majority of patients with ACS.

During a median follow-up of 1.2 years, the incidence of MACEs after discharge from hospital was lower than in similar earlier studies.

Among the baseline potential clinical predictors, presence of treated diabetes, LV ejection fraction, history of AMI, Charlson comorbidity index, and beta-blocker prescription at discharge demonstrated the most significant independent effect on the development of MACEs. At 6 months, the number of uncontrolled TRFs significantly affected the prognosis of MACEs.

We did not find a relationship between the initial quantitative characteristics of carotid/subclavian AP and their 6-month changes with MACE probability. At the same time, we demonstrated a prognostic significance of AP GSM assessed both at baseline and at 6 months which was independent of clinical predictors and risk factor control. According to our knowledge, this is the first confirmation of this variable’s independent prognostic role after ACS. Our results support the routine use of ultrasound imaging of the carotid/subclavian AP with the calculation of standardized GSM to improve the MACE risk prediction. This result is also hypothesis-generating, and the utility of longitudinal carotid GSM evaluation for the assessment of secondary prevention adequacy after ACS may be the subject of further studies.

## Figures and Tables

**Table 1:** Baseline characteristics of study participants by MACE occurrence.

	Total	Without MACE	With MACE
**History and clinical characteristics**			
Male			
No	98 (30.4%)	86 (30.3%)	12 (31.6%)
Yes	224 (69.6%)	198 (69.7%)	26 (68.4%)
Age (years)	65 [59; 73]	66 [59; 73]	62 [59; 72]
Body mass index	28 [26; 31]	28 [26; 31]	29 [26; 31]
Smoking			
Never	171 (53.1%)	155 (54.6%)	16 (42.1%)
Smokes now/previously	151 (46.9%)	129 (45.4%)	22 (57.9%)
Arterial hypertension			
No	129 (40.2%)	114 (40.3%)	15 (39.5%)
Yes	192 (59.8%)	169 (59.7%)	23 (60.5%)
Diabetes			
No/on diet	255 (79.2%)	234 (82.4%)	21 (55.3%)
Oral medication/insulin	67 (20.8%)	50 (17.6%)	17 (44.7%)
Peripheral arterial disease			
No	296 (91.9%)	261 (91.9%)	35 (92.1%)
Yes	26 (8.1%)	23 (8.1%)	3 (7.9%)
History of myocardial infarction			
No	232 (72.0%)	210 (73.9%)	22 (57.9%)
Yes	90 (28.0%)	74 (26.1%)	16 (42.1%)
History of stroke			
No	297 (92.2%)	265 (93.3%)	32 (84.2%)
Yes	25 (7.8%)	19 (6.7%)	6 (15.8%)
Moderate-intensity physical activity (min/week)	200 [40; 420]	205 [50; 415]	165 [30; 420]
Vigorous physical activity (min/week)	20 [0; 60]	20 [5; 60]	20 [0; 50]
Physical activity as per Guidelines			
No	121 (37.6%)	105 (37.0%)	16 (42.1%)
Yes	201 (62.4%)	179 (63.0%)	22 (57.9%)
**Clinical variables related to ACS**			
Time from onset of pain to PCI (min)	340 [180; 630]	300 [180; 612]	430 [200; 720]
Heart rate on admission (in min)	72 [65; 80]	72 [65; 80]	72 [67; 80]
Heart rate at discharge (in min)	66 [61; 71]	65 [61; 71]	66 [62; 73]
SBP (mmHg)	140 [130; 150]	140 [130; 150]	140 [125; 155]
Hemoglobin (g/L)	141 [130; 151]	142 [130; 151]	136 [129; 146]
Creatinine (µmol/L)	92 [80; 108]	94 [81; 108]	84 [73; 102]
eGFR * (mL/min/1.73 m^2^)	69 [56; 82]	69 [55; 82]	74 [58; 89]
LDL-C (mmol/L)	3 [2; 4]	3 [2; 4]	3 [2; 4]
LDL-C/HDL-C	3 [2; 4]	3 [2; 4]	3 [2; 4]
ACS type			
STEMI	137 (42.7%)	124 (43.8%)	13 (34.2%)
NSTEMI	94 (29.3%)	78 (27.6%)	16 (42.1%)
UA	90 (28.0%)	81 (28.6%)	9 (23.7%)
Killip class			
1	254 (89.4%)	34 (89.5%)	288 (89.4%)
≥2	30 (10.6%)	4 (10.5%)	34 (10.6%)
Number of ECG leads with ST elevation/depression	3 [0; 5]	3 [0; 5]	3 [0; 4]
Area of LV dyssynergy by echocardiography (%)	12 [0; 25]	12 [0; 25]	18 [6; 25]
Number of segments with WMA	2 [0; 4]	2 [0; 4]	3 [1; 4]
WMSI	1 [0; 2]	1 [0; 2]	1 [1; 2]
LV ejection fraction (%)	61 [54; 66]	62 [55; 66]	59 [52; 64]
Presence of LM stenosis			
No	285 (88.5%)	252 (88.7%)	33 (86.8%)
Yes	37 (11.5%)	32 (11.3%)	5 (13.2%)
Three-vessel coronary artery disease			
No	229 (71.3%)	207 (73.1%)	22 (57.9%)
Yes	92 (28.7%)	76 (26.9%)	16 (42.1%)
SYNTAX score	14 (8–22)	14 (8–21)	14 (10–26)
Troponin (max, pg/mL)	647 [104; 3700]	647 [103; 3947]	604 [109; 2619]
Charlson comorbidity index	4 [2; 5]	4 [2; 5]	4 [3; 6]
PRECISE-DAPT score	18 [12; 27]	18 [12; 27]	14 [10; 21]
**Characteristics of the carotid/subclavian AP**			
Hsum (mm)	7 [4; 11]	8 [4; 12]	7 [4; 10]
TPA (mm^2^)	50 [27; 89]	51 [28; 90]	44 [22; 73]
Standardized GSM	94 [72; 122]	95 [73; 124]	83 [58; 108]
Standardized GSM < 81			
No	178 (66.9%)	162 (69.2%)	16 (50.0%)
Yes	88 (33.1%)	72 (30.8%)	16 (50.0%)
**Treatment at hospital discharge**			
Complete revascularization			
No	186 (57.8%)	159 (56.0%)	27 (71.1%)
Yes	136 (42.2%)	125 (44.0%)	11 (28.9%)
High-intensity statins			
No	15 (4.7%)	14 (4.9%)	1 (2.6%)
Yes	307 (95.3%)	270 (95.1%)	37 (97.4%)
Ezetimibe			
No	299 (92.9%)	262 (92.3%)	37 (97.4%)
Yes	23 (7.1%)	22 (7.7%)	1 (2.6%)
DATT			
No	8 (2.5%)	6 (2.1%)	2 (5.3%)
Yes	314 (97.5%)	278 (97.9%)	36 (94.7%)
ACE inhibitors/ARBs			
No	10 (3.1%)	10 (3.5%)	0 (0.0%)
Yes	312 (96.9%)	274 (96.5%)	38 (100.0%)
Beta-blockers			
No	25 (7.8%)	19 (6.7%)	6 (15.8%)
Yes	297 (2.2%)	265 (3.3%)	32 (84.2%)

MACE—major adverse cardiovascular event; ACS—acute coronary syndrome; PCI—percutaneous coronary intervention; SBP—systolic blood pressure; LDL-C—low-density lipoprotein cholesterol; HDL-C—high-density lipoprotein cholesterol; STEMI—ST elevation myocardial infarction; NSTEMI—non-ST elevation myocardial infarction; UA—unstable angina; ECG—electrocardiogram; LV—left ventricular; WMA—wall motion abnormality, WMSI—wall motion score index; LM stenosis—left main stenosis; AP—atherosclerotic plaque; TPA—total plaque area; GSM—gray scale median; DATT—*double* antithrombotic *therapy; ACE*—angiotensin-converting enzyme; ARBs—angiotensin 2 receptor blockers; *—by CKD-EPI formula.

**Table 2:** Characteristics of participants at 6 months after primary ACS depending on the occurrence of MACEs.

	Total	Without MACE	With MACE
**Risk factor control**			
SBP (mm Hg)	125 [120; 130]	125 [120; 130]	130 [120; 135]
DBP (mm Hg)	75 [70; 80]	75 [70; 80]	80 [70; 80]
Target blood pressure			
No	56 (18.6%)	48 (18.0%)	8 (22.9%)
Yes	245 (81.4%)	218 (82.0%)	27 (77.1%)
LDL-C (mmol/L)	2 [2; 2]	2 [2; 2]	2 [2; 2]
LDL-C/HDL-C	2 [1; 2]	2 [1; 2]	2 [1; 2]
Target LDL-C *			
No	198 (87.6%)	175 (86.6%)	23 (95.8%)
Yes	28 (12.4%)	27 (13.4%)	1 (4.2%)
Current smoker			
No	213 (73.2%)	188 (72.9%)	25 (75.8%)
Yes	78 (26.8%)	70 (27.1%)	8 (24.2%)
Physical activity as per Guidelines			
No	109 (37.2%)	94 (36.4%)	15 (42.9%)
Yes	184 (62.8%)	164 (63.6%)	20 (57.1%)
Number of uncontrolled risk factors			
0–2	181 (80.8%)	167 (83.5%)	14 (58.3%)
3–4	43 (19.2%)	33 (16.5%)	10 (41.7%)
**Characteristics of the carotid/subclavian AP**			
Hsum (mm)	7 [4; 11]	8 [4; 11]	6 [4; 9]
TPA (mm^2^)	51 [27; 88]	52 [28; 91]	43 [27; 64]
Standardized GSM	94 [73; 119]	96 [76; 119]	80 [58; 109]
Standardized GSM < 81			
No			
Yes			
Hsum compared to baseline			
No change	166 (75.1%)	147 (75.0%)	19 (76.0%)
Decreased	18 (8.1%)	15 (7.7%)	3 (12.0%)
Increased	37 (16.7%)	34 (17.3%)	3 (12.0%)
TPA compared to baseline			
No change	133 (60.2%)	118 (60.2%)	15 (60.0%)
Decreased	23 (10.4%)	21 (10.7%)	2 (8.0%)
Increased	65 (29.4%)	57 (29.1%)	8 (32.0%)
Standardized GSM compared to baseline			
No change	115 (62.8%)	102 (63.7%)	13 (56.5%)
Decreased	9 (4.9%)	8 (5.0%)	1 (4.3%)
Increased	59 (32.2%)	50 (31.2%)	9 (39.1%)
**Treatment**			
High-intensity statins			
No	29 (9.8%)	26 (10.0%)	3 (8.6%)
Yes	266 (90.2%)	234 (90.0%)	32 (91.4%)
Ezetimibe			
No	260 (88.1%)	228 (87.7%)	32 (91.4%)
Yes	35 (11.9%)	32 (12.3%)	3 (8.6%)
DATT			
No	17 (5.8%)	15 (5.8%)	2 (5.7%)
Yes	278 (94.2%)	245 (94.2%)	33 (94.3%)
ACE inhibitors/ARBs			
No	15 (5.1%)	14 (5.4%)	1 (2.9%)
Yes	280 (94.9%)	246 (94.6%)	34 (97.1%)
Beta-blockers			
No	27 (9.2%)	22 (8.5%)	5 (14.3%)
Yes	268 (90.8%)	238 (91.5%)	30 (85.7%)

MACE—major adverse cardiovascular event; SBP—systolic blood pressure; DBP—diastolic blood pressure; LDL-C—low-density lipoprotein cholesterol; HDL-C—high-density lipoprotein cholesterol; TPA—total plaque area; GSM—gray scale median; DATT—*double* antithrombotic *therapy; ACE*—angiotensin-converting enzyme; ARBs—angiotensin 2 receptor blockers; *—<1.4 mmol/L *and* a 50% reduction from baseline.

**Table 3:** Variables showing significant/borderline nonadjusted effects on MACEs, based on univariate Cox regression.

Baseline Variables	HR	95% CI	*p*
Diabetes			
No/diet			
Oral medication/insulin	3.09	1.63–5.87	0.001
History of myocardial infarction			
No			
Yes	2.11	1.11–4.03	0.023
Charlson comorbidity index	1.27	1.08–1.49	0.004
LV ejection fraction (%)	0.86	0.75–0.98	0.026
Three-vessel coronary artery disease			
No			
Yes	2.11	1.10–4.02	0.024
Standardized GSM < 81			
No			
Yes	1.91	0.95–3.87	0.071
Heart rate at discharge (bpm)	1.05	1.01–1.08	0.007
Beta-blockers at discharge			
No			
Yes	0.41	0.17–0.98	0.045
**Variables assessed after 6 months**			
Uncontrolled risk factors			
0–2			
3–4	2.91	1.28–6.58	0.010
Standardized GSM < 81			
No			
Yes	2.39	1.04–5.48	0.040

HR—hazard ratio; CI—confidence interval; LV—left ventricular; GSM—gray scale median.

**Table 4:** Multivariable Cox regression analysis of MACE development after ACS: baseline variables.

Predictors	HR	Original Model		After Internal Validation	
		95% CI	*p*	95% CI	*p*
**Model 1**					
Diabetes	2.22	1.08–4.57	0.030	1.04–4.74	0.039
Standardized GSM < 81	2.06	1.02–4.19	0.045	1.03–4.15	0.042
Decrease in LV ejection fraction (by every 5% from 60%)	1.22	1.03–1.46	0.023	1.02–1.47	0.030
**Model 2**					
Diabetes	2.28	1.11–4.68	0.025	1.04–4.99	0.039
Standardized GSM < 81	1.98	0.98–4.03	0.058	1.00–3.94	0.038
Decrease in LV ejection fraction (by every 5% from 60%)	1.24	1.04–1.48	0.015	1.05–1.46	0.011
No beta-blockers at discharge	3.24	1.32–7.97	0.011	1.07–9.82	0.038
**Model 3**					
Charlson comorbidity index	1.24	1.05–1.48	0.014	1.03–1.50	0.021
Standardized GSM < 81	2.01	0.99–4.08	0.052	1.05–3.85	0.034
Decrease in LV ejection fraction by every 5% (from 60%)	1.22	1.03–1.44	0.022	1.00–1.49	0.050
**Model 4**					
Charlson comorbidity index	1.27	1.06–1.52	0.008	1.05–1.54	0.013
Standardized GSM < 81	1.97	0.97–3.99	0.061	0.90–4.31	0.091
Decrease in LV ejection fraction (by every 5% from 60%)	1.23	1.04–1.46	0.015	1.03–1.48	0.025
No beta-blockers at discharge	3.46	1.40–8.55	0.007	1.27–9.40	0.015

HR—hazard ratio; CI—confidence interval; GSM—gray scale median; LV—left ventricular.

**Table 5:** Multivariate Cox regression analysis of MACE development after ACS: variables assessed at 6 months and the most significant baseline variables.

Predictors	HR	Original Model		After Internal Validation	
		95% CI	*p*	95% CI	*p*
**Model 5**					
Diabetes	3.80	1.27–11.35	0.017	1.33–10.81	0.012
Standardized GSM < 81 at 6 months	3.77	1.43–9.92	0.007	1.16–12.20	0.027
≥3 uncorrected risk factors at 6 months	3.11	1.17–8.25	0.023	1.15–8.42	0.026
**Model 6**					
Charlson comorbidity index	1.31	1.03–1.68	0.030	1.01–1.70	0.038
Standardized GSM < 81 at 6 months	4.03	1.52–10.65	0.005	1.29–12.54	0.016
≥3 uncorrected risk factors at 6 months	2.27	0.87–5.89	0.092	0.79–6.49	0.126

HR—hazard ratio; CI—confidence interval; GSM—gray scale median.

**Table 6:** Baseline cumulative MACE risks at 180, 360, and 720 days after ACS.

Calculator	Baseline Predictor Values	180 Days	360 Days	720 Days
(based on models 1–2)	DM—no or on diet LV ejection fraction—60% Baseline standardized GSM < 81—no No beta-blockers at discharge—no	2.05%	3.35%	11.19%
2 (based on models 3–4)	Charlson comorbidity index—0 LV ejection fraction—60% Baseline standardized GSM < 81—no No beta-blockers at discharge—no	0.93%	1.52%	5.29%

DM—diabetes mellitus; LV—left ventricular; GSM—gray scale median.

## Data Availability

Data are available on request due to restrictions (dataset contains personal patient data).

## Supplementary Materials

The following supporting information can be downloaded at: https://www.mdpi.com/article/10.3390/life15030431/s1, Figure S1: Representative cases of carotid plaques with different GSM values; Figure S2: Goodness-of-fit graphs for models 1-4; Figure S3: Goodness-of-fit graphs for models 5 and 6. Table S1: Multivariate regression models for baseline variables. Sets of predictors for stepwise selection and final sets of predictors; Table S2: Multivariate regression models for variables evaluated at 6 months and most important baseline variables. Sets of predictors for stepwise selection and final sets of predictors.
