# Supplementary material for: The Value of Clinical Variables and the Potential of Longitudinal Ultrasound Carotid Plaque Assessment in Major Adverse Cardiovascular Event Prediction After Uncomplicated Acute Coronary Syndrome
Source: Life (Basel). 2025 Mar 9;15(3):431. doi: 10.3390/life15030431 (PMC11943730; doi:10.3390/life15030431)
Supplement: Supplementary file 1 [file life-15-00431-s001.zip › life-3505803-supplementary.pdf]

Fig. S1. Representative cases of carotid plaques with different GSM values.

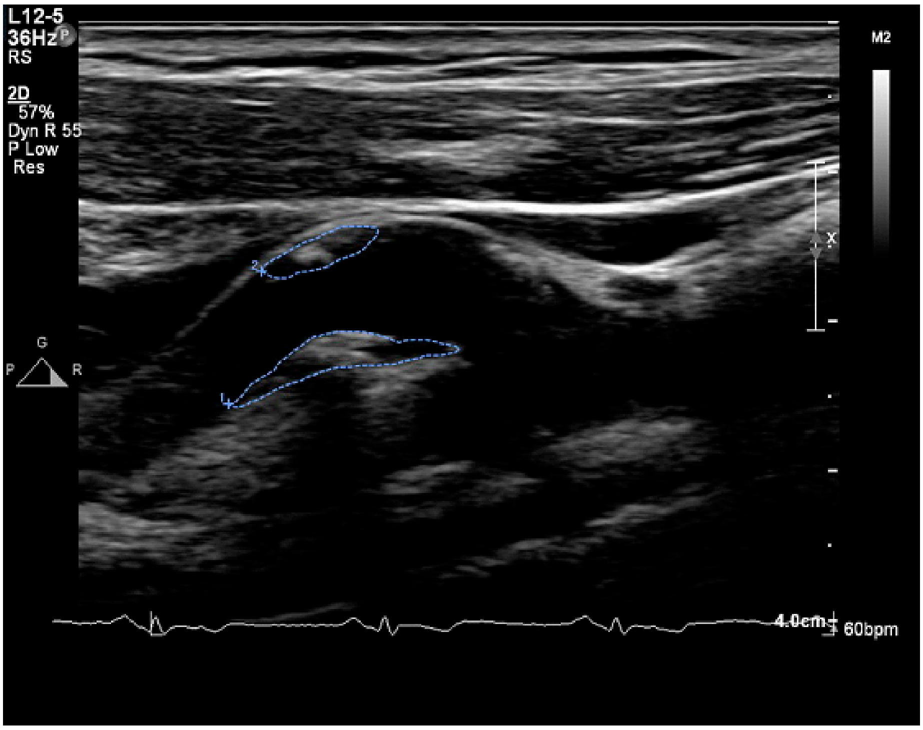

Two heterogeneous atherosclerotic plaques in the origin of left ICA. Plaques are traced to measure their areas. The near wall plaque GSM was calculated to be 66, far wall plaque GSM was 104.

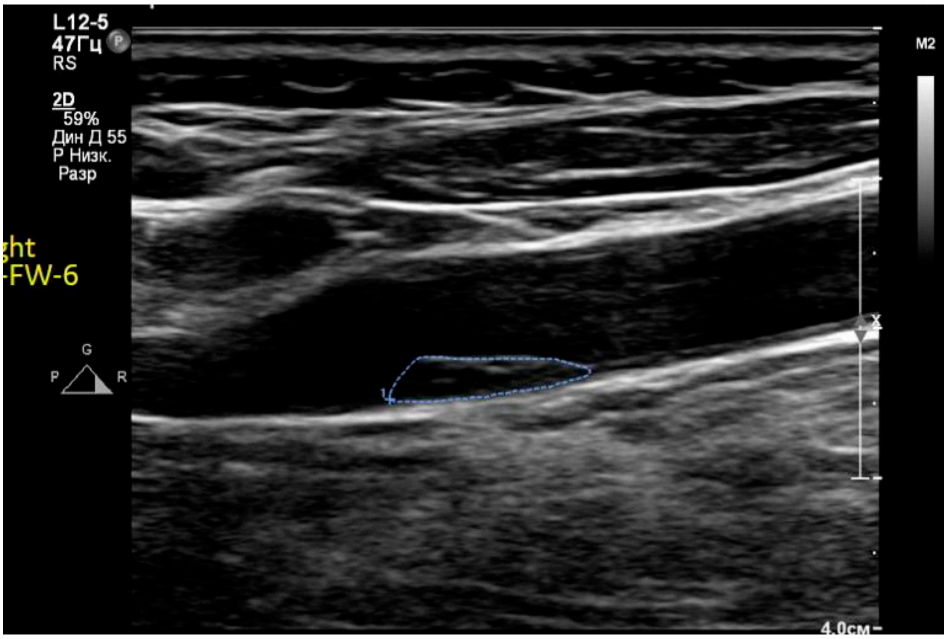

Predominantly hypoechoic plaque in the right carotid bifurcation and origin of ICA. Plaque is traced to measure its area. Calculated GSM was 28.

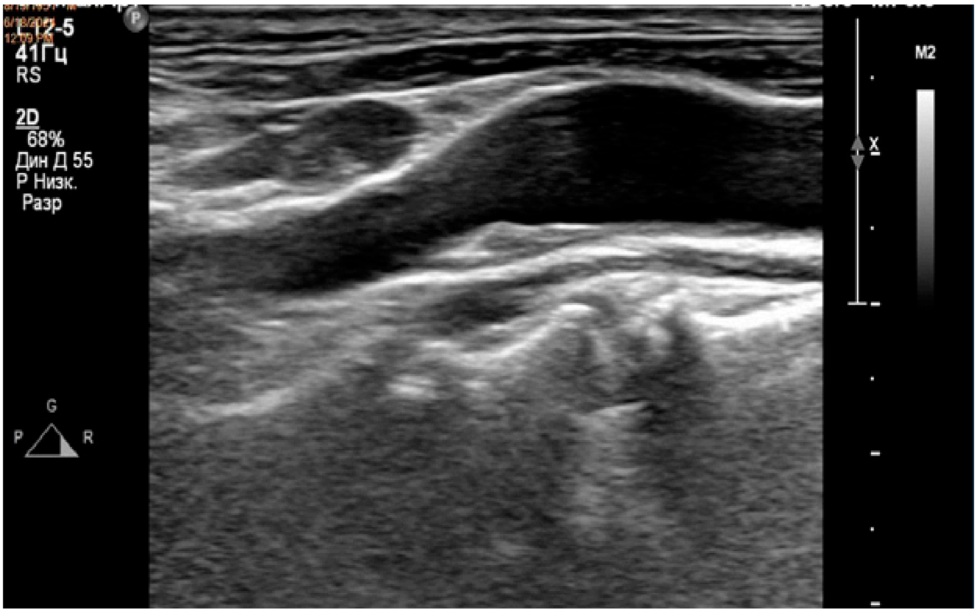

Heterogeneous predominantly echodense plaque in proximal ICA, GSM=111

ICA – internal carotid artery; GSM - gray scale median

Table S1. Multivariate regression models for baseline variables. Sets of predictors for stepwise selection and final sets of predictors.

Model	Set of predictors for stepwise selection	Final set of predictors	AIC	BIC	Harrell's C-index, %
1	- Diabetes<br>- History of myocardial infarction<br>- History of stroke<br>- Heart rate at discharge<br>- Decrease in LV ejection fraction (by every 5% from 60%)<br>- three-vessel coronary artery disease<br>- Standardized GSM < 81<br>- complete revascularization at discharge	- Diabetes<br>- Standardized GSM < 81<br>- Decrease in LV ejection fraction (by every 5% from 60%)	303.23	313.97	68.7
2	- diabetes<br>- History of myocardial infarction<br>- History of stroke<br>- No beta-blockers at discharge<br>- LV ejection fraction (%)<br>- three-vessel coronary artery disease<br>- Standardized GSM < 81<br>- complete revascularization at discharge	- diabetes<br>- LV ejection fraction (%)<br>- Standardized GSM < 81<br>- No beta-blockers at discharge	299.86	314.17	70.3
3	- Charlson comorbidity index<br>- Heart rate at discharge<br>- LV ejection fraction (%)<br>- three-vessel coronary artery disease<br>- Standardized GSM < 81<br>- complete revascularization at discharge	- Charlson comorbidity index<br>- LV ejection fraction (%)<br>- Standardized GSM < 81	302.10	312.84	69.1
4	- Charlson comorbidity index<br>- No beta-blockers at discharge<br>- LV ejection fraction (%)	- Charlson comorbidity index<br>- No beta-blockers at discharge	298.25	312.55	69.8

	- three-vessel coronary artery disease<br>- Standardized GSM < 81<br>- complete revascularization at discharge	- LV ejection fraction (%)<br>- Standardized GSM < 81			

GSM - gray scale median; LV – left ventricular.

Calibration of models 1-4

The agreement between the predicted and observed values (goodness-of-fit) was assessed visually (Fig. S1) and with the Groennesby and Borgan test.

For the presented models, the graphical discrepancy between the predicted and observed risks is observed only at the end of the curve, which is probably due to the small number of observations. The Groennesby and Borgan test results and visual assessment of the goodness-of-fit plots indicate good calibration of the models.

Fig. S2. Goodness-of-fit graphs for models 1-4.

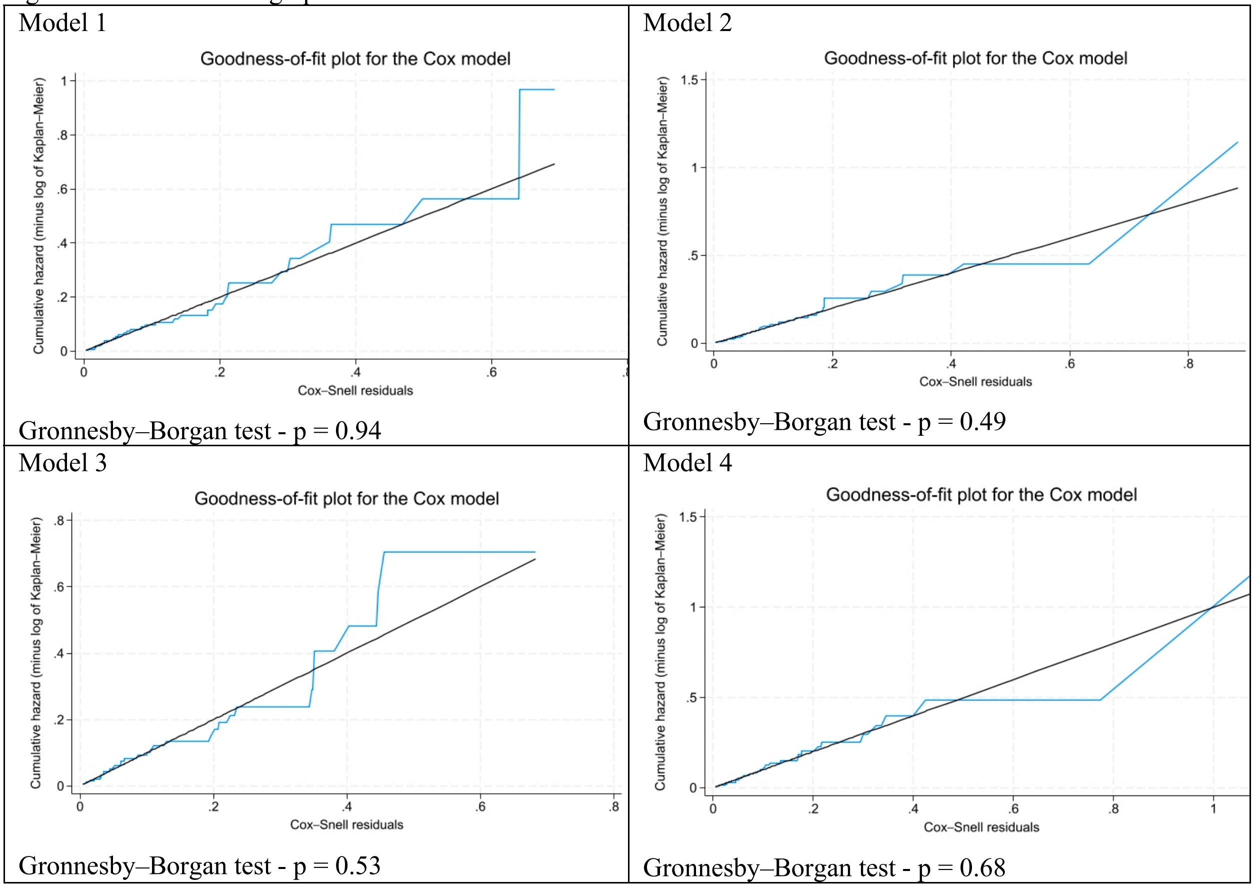

Table S2. Multivariate regression models for variables evaluated at 6 months and most important baseline variables. Sets of predictors for stepwise selection and final sets of predictors.

Model	Set of predictors for stepwise selection	Final set of predictors	AIC	BIC	Harrell's C-index, %
5	- diabetes<br>- LV ejection fraction (%)<br>- Standardized GSM < 81<br>- ≥3 uncorrected risk factors at 6 months	- diabetes<br>- Standardized GSM < 81<br>- ≥3 uncorrected risk factors at 6 months	147.78	157.21	70.8
6	- Charlson comorbidity index<br>- LV ejection fraction (%)<br>- Standardized GSM < 81<br>- ≥3 uncorrected risk factors at 6 months	- Charlson comorbidity index<br>- Standardized GSM < 81<br>- ≥3 uncorrected risk factors at 6 months	148.58	158.01	74.4

GSM - gray scale median; LV – left ventricular.

Fig. S3. Goodness-of-fit graphs for models 5 and 6

Model 5

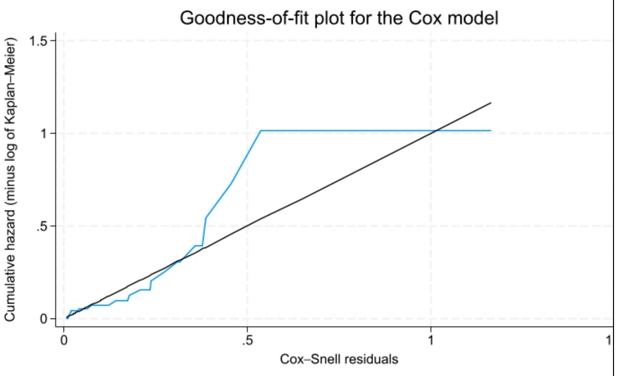

Gronesby-Borgan test - p = 0.0195

Model 6

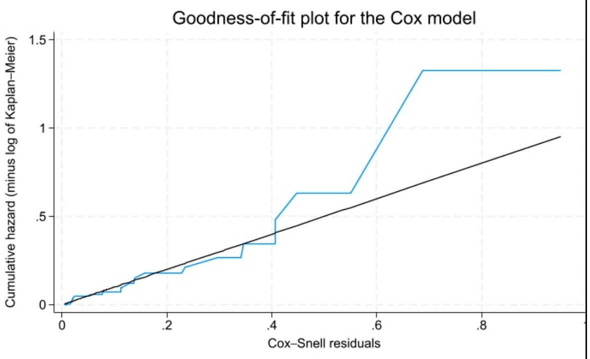

Gronnesby–Borgan test - p = 0.0870
